# Research and Verification of Convolutional Neural Network Lightweight in BCI

**DOI:** 10.1155/2020/5916818

**Published:** 2020-08-01

**Authors:** Shipu Xu, Runlong Li, Yunsheng Wang, Yong Liu, Wenwen Hu, Yingjing Wu, Chenxi Zhang, Chang Liu, Chao Ma

**Affiliations:** ^1^Department of Software Engineering, Tongji University, Shanghai 201804, China; ^2^Agricultural Information Institutes of Science and Technology, Shanghai Academy of Agricultural Sciences, Shanghai 201403, China; ^3^Department of Railway Transportation, Shanghai Institute of Technology, Shanghai 201418, China; ^4^School of Information Engineering, Nanchang Hangkong University, Nanchang Jiangxi 330038, China

## Abstract

With the increasing of depth and complexity of the convolutional neural network, parameter dimensionality and volume of computing have greatly restricted its applications. Based on the SqueezeNet network structure, this study introduces a block convolution and uses channel shuffle between blocks to alleviate the information jam. The method is aimed at reducing the dimensionality of parameters of in an original network structure and improving the efficiency of network operation. The verification performance of the ORL dataset shows that the classification accuracy and convergence efficiency are not reduced or even slightly improved when the network parameters are reduced, which supports the validity of block convolution in structure lightweight. Moreover, using a classic CIFAR-10 dataset, this network decreases parameter dimensionality while accelerating computational processing, with excellent convergence stability and efficiency when the network accuracy is only reduced by 1.3%.

## 1. Introduction

In the 5G era, with the development of emerging technologies such as the Internet of Things and big data, related applications in smart terminals are becoming more and more widespread. As a support for these intelligent applications, brain-computer-interface (BCI) technology plays an essential role in intelligent identification, classification, and computing. Our work mainly focuses on the intelligent recognition of images and videos, which is an indispensable intelligent application in life.

Since the publication of the 2006 Hinton research [1], deep learning algorithms have evolved rapidly. Based on the traditional artificial neural network (ANN) and the processing power of modern computers, it has achieved remarkable results in image processing, speech recognition, and scene analysis. The processing power and algorithm performance of complex problems have been greatly improved, which has attracted widespread attention from academia and industry. The idea of deep learning can be summarized as unsupervised learning from bottom to top and parameter adjustment from top to bottom. Its adjustment process is based on the traditional BP algorithm. Typical deep learning algorithm models mainly include encoder, deep belief networks (DBN), and convolutional neural networks.

**Figure 1:**
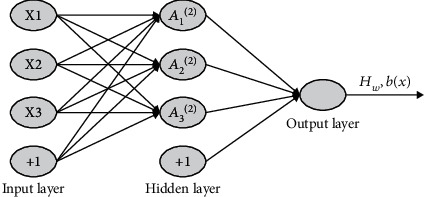
Traditional ANN model.

The development of ANN can be tracked back to the 1940s, and its development process is roughly divided into three stages. The first stage was the submission of the neuron model and learning rules from 1947-1969, such as perceptron, HEBB learning rules, binary neuron model (MP model), etc. The second stage is the HNN neural network model introduced by Professor Hopfield in 1982 by introducing the concept of Energy Function. The third stage is the classic back-propagation algorithm proposed by Professor Rumelhart in 1986. This algorithm is now known as the BP algorithm [2]. A typical three-layer ANN model is shown in Figure 1.

One of the benefits of deep learning frameworks in image recognition is that they do not need the traditional classification algorithms. It requires a lot of artificial processing of image features.

It is an adaptive algorithm. Through multilayer convolution and a nonlinear activation function, the algorithm classifies and regresses all image features through MLP [3]. It has the characteristics of migration invariance, scale invariance, and radiance image brightness. The overall CNN model starts with a convolutional layer. A convolution layer is a series of feature filter pairs (filter set), which contains multiple convolution kernels, and the output is feature maps. Then, convolution results are linearly modified; using RELU [4] function. After the convolution, a pooling layer is usually added. Generally, there is a mean pooling layer and a maximum pooling layer to compress the image.

Many classic deep learning network architectures have been developed based on the ILSVRC platform, such as AlexNet [5], ZFNet [6], VGGNet [7], GoogleNet [8], and ResNet. VGGNet is an improved framework based on the 1000-class image classification and localization model using the image-Net model. Due to the characteristics of neural networks, in order to obtain high accuracy, existing pipelines tend to increase the depth and complexity of their networks continuously. The number of internal parameters and the nonlinear mapping tends to be huge, which makes the deep network structure perform well in competition and data reflection. However, real-world applications are often constrained by storage space, computing power, and computing speed of the terminals. For example, in practical applications such as automatic driving, face recognition on mobile phones, video classification, etc., learning results are often demanded in milliseconds. Additionally, these devices often have limited processor performance with no prior trainings in the lab. Therefore, the practicality of CNN could be limited.

Two lines of work have been proposed to make deep learning networks applicable to daily lives. One is to improve hardware. The other is to improve the computing power of mobile terminals and to improve network structures, with a goal to minimize the training time and the amount of data required without affecting the accuracy. The development speed of the hardware is relatively slow, and its update iteration is far behind the speed required for the evolution of the network structure. Therefore, reducing the calculation parameters and calculation complexity of traditional network frameworks has gained the most research interests in deep learning.

## 2. Related Works

Since the discovery of electrodes that can be used to collect EEG signals from the subcortex in the 1930s, research on EEG signals has provided experimental tools to decode neural substrates that are associated with thoughts and feelings of study subjects. With the rapid development of pattern recognition algorithms, ANNs, and deep learning frameworks, research on brain-computer interface (BCI) systems is in full swing.

BCI system-evoked potential collection methods include nonimplantable electroencephalogram (EEG) [7], implantable electroencephalogram (EcoG) [8], and functional magnetic resonance imaging (functional magnetic resonance imaging) [9]. The acquisition of nonimplantable EEG signals will not cause damage to the cerebral cortex. As a convenient, simple, and low-cost method, it has been widely used in the field of brain-computer interface system research.

**Figure 2:**
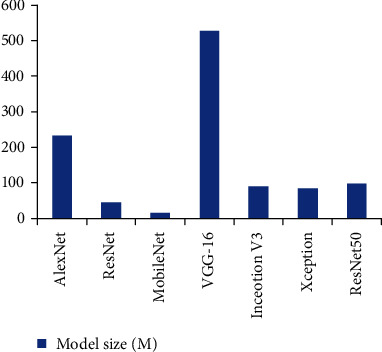
Comparison of famous model quality.

The five-layer CNN has AlexNet and its optimized networks such as ZF, VGG, GoogleNet, ResNet, and DenseNet. Their performance is gradually improving, but the amount of parameters is also increasing. See Figure 2 for a comparison of the performance and quality of some of the more popular network models recently. It can be seen that the quality of these volume computer networks is mostly in the tens to hundreds of megabytes.

UC Berkeley proposed the SqueezeNet convolutional network model in 2016. This model can reduce these tens of megabytes and hundreds of megabytes of network structure to about 4.6 megabytes without affecting accuracy. This paper proposes three improvement strategies for SqueezeNet's core module, Fire Module. The first strategy is to improve on the dense 1 × 1 convolution [9] kernel by using 1 × 1 grouping convolution to reduce the number of calculations. This strategy can also solve the problem of noncirculation of channel information in grouped convolution. The second strategy is to add channel shuffle [10] (cross grouping) operation. This strategy reorganizes the different feature maps after grouping convolutions, so that the next grouping convolutions come from different groups, making information flow in various groups. The third strategy is to adjust at the SoftMax layer and use SoftMax Loss [11] and Center Loss [12] to monitor training jointly. This training can be used to compensate for the high similarity image recognition effect.

In order to research the lightweight of CNNs in BCI, in 2015, Professor He Kaiming introduced a new structure of deep residual neural networks [13, 14]. The CNN trained on this structure has a depth from the AlexNet8 layer to the VGG19 layer to the ResNet152 layer and can converge and train regularly. ResNet won the championship with an accuracy of 16% over the second place in the ImageNet detection task and surpassed the second place by 27% in the ImageNet positioning task.

## 3. Improvements and Design of Fire Module

### 3.1. Improvements of Fire Module

**Figure 3:**
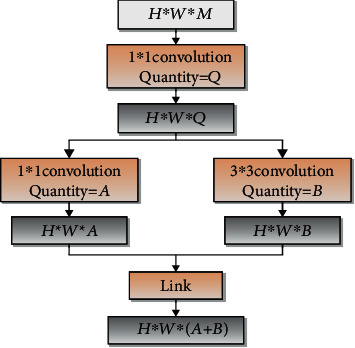
Composition of Fire Module

As shown in Figure 3, the core module of the SqueezeNet network model is the Fire Module. It consists of the Squeeze layer and the Expand layer. The Squeeze layer is composed of a 1 × 1 convolution kernel. The 1 × 1 convolution kernel can change or reduce the number of channels when the model is input by changing its own convolution kernel number. Finally, the purpose of reducing the number of parameters and computational complexity is achieved. The Expand layer consists of a 1 × 1 convolution kernel and a 3 × 3 convolution kernel. Because the 1 × 1 convolution calculation in the convolution operation accounts for most of the entire module, the calculation complexity is still high. This paper replaces the original 1 × 1 conventional convolution kernel with a 1 × 1 packet convolution on the basis of Fire-Module. In addition, batch normalization (BN) is used for the input of the model to speed up the training and convergence process and improve the classification accuracy. Finally, the strategy cascades improved Fire Modules.

### 3.2. Grouping Convolutional Appointments

**Figure 4:**
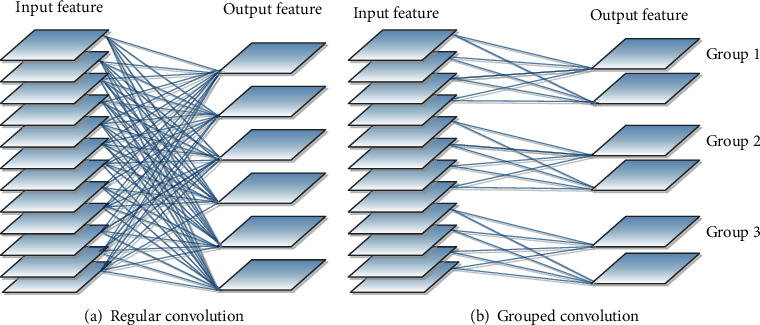
Regular convolution and grouped convolution.

The comparison between grouped convolution and regular convolution is shown in Figure 4. The traditional convolution is shown in Figure 1. The convolution kernel is completely converted for training. In the grouping convolution, the convolution kernel is divided into *N* (*N* = 3 in the figure) parts, and the input dimension is *D*_in_/*N*. In the grouping convolution, the convolution kernel corresponds to the input [:, :0 : *D*_in_/*N*] dimension part for convolution operation. The second set of convolution kernels and the input [:, :*D*_in_/*N* : 2*D*_in_/*N*] dimensions are used for convolution. According to this, it is concluded that the output after the convolution operation of each group has become a convolution kernel in the *D*_0_/*N* dimension. And each set of input and output operations is independent convolution operations. The input is convolved only with the current grouping convolution kernel and not with other grouping convolution kernels. After all the grouping convolutions are completed, the outputs of all *D*_0_/*N* dimensions are superimposed to obtain the complete output of the final grouping convolution.

It is clear from Table 1 that the comparison of the conventional convolution parameters of the grouped convolution kernel is directly proportional to their ratio and number of groups. When the input size is *W*_1_ (width), *H*_1_ (height), and *C*_1_ (size), assuming a *C*_2_ convolution kernel and the size of the convolution kernel is *h* × *w*, the calculations in the above table can be regarded as grouping convolution and regular convolution calculations and parameters. It can be concluded that the use of grouped convolution can significantly reduce the number of parameters and the calculation of the entire model.

### 3.3. Channel Shuffle

Because the input is a whole, the output after convolution is also mapped to the whole of the input. In the grouping convolution, the training of each grouping convolution is performed independently for each channel. This is equivalent to dividing the overall input into many independent parts for convolution. Therefore, the independent operation between each group will cause the information of each group channel to flow. To strengthen the information exchange between each packet, this article adds channel shuffle operation on this basis.

**Figure 5:**
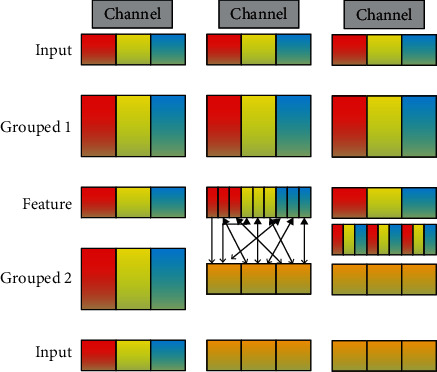
The principle of group convolution. (a) The channels are grouped into 3 groups, and there is no communication between different groups of feature maps. (b) Reconstructed feature maps. (c) Channel shuffle after convolution

As shown in Figure 5, cross scramble the grouped features to form a new feature and input it into the next round of convolution operations. This allows the input of the grouping convolution to come from different groups and allows the information between different independent groups to circulate.

### 3.4. Improvement of Loss Function SoftMax

SqueezeNet uses a conventional SoftMax classifier, and the SoftMax function is a finite discrete probability distribution function. The SoftMax function, as shown in

(1) $$\begin{array}{c} L_{S}=-\overset{m}{\underset{i=1}{\sum }}\log\frac{e^{w_{y_{i}}^{T}x_{i}}+b_{y_{i}}}{\sum_{j=1}^{n}e_{y_{i}}^{w^{t}}x_{i}+b_{y_{i}}}. \end{array}$$

For probabilistic multiclassification problems, it is simple and effective. However, the high similarity and features of human facial pictures are not apparent, and the class spacing of their features is often substantial. The intraclass distance is likely to be larger than the interclass distance. This will result in a lower recognition rate under complex face pictures. The Center loss function is shown in

(2) $$\begin{array}{c} L_{C}=\frac{1}{2}\overset{m}{\underset{i=1}{\sum }}{\left\|x_{i}-c_{y_{i}}\right\|}_{2}^{2}. \end{array}$$

The mixed loss function is shown in

(3) $$\begin{array}{c} L=L_{s}+\gamma L. \end{array}$$

In Equation (3), *x*_*i*_ represents the features before the fully connected layer, and  *c*_*yi*_ represents the center of the  *y*_*i*_  branch. The difference of Center Loss is that it adds a center to each class branch and, on this basis, increases the distance between other class branches and the center. So it makes the gap between classes smaller and the distance between classes larger. These features are more useful for classifying some complex images. Based on this, the SoftMax classification layer of SqueezeNet is improved to become a joint classification of SoftMax-Center Loss to reduce class spacing, which makes it more useful to recognize complex and similar face models.

### 3.5. NVMNet

**Figure 6:**
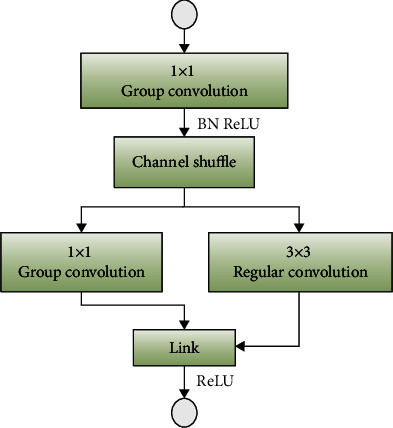
Improved NVM.

The Fire Module improved based on the above method is shown in Figure 6. This article is named the NVM (New Visual Module). Based on this, the NVMNet (New Visual Module Net) structure is established. Compared with the previous Fire Module, the NVM is also composed of a compression layer and an expansion layer. This paper replaces the last 1 × 1 conventional convolution with a grouped convolution to reach the model reduction. It reduced the number of input channels by 1 × 1 grouping convolution in the compression layer before and added batch normalization after the 1 × 1 convolution to speed up the training process. Then, channel shuffle allows the data to circulate the packet training information in different channels.

According to the hyperparameters of the previous module, the grouping number *g* of the grouping convolution of the Squeeze layer and the Expand layer, the number of convolution kernels *h* of the layer Squeeze, the number of convolution kernels w of the convolution layer, and the number of convolution kernels *n* of the expansion layer are set. Among them, *h* = *m*, *h* < *n*, and *w* < *n*.

The dimensions and dimensions of the input and output of the entire model are the same. This article refers to the structure of the SqueezeNet model by linking the improved NVM. And add a pooling layer and two NVM to the fabric. Besides, add the SoftMax-Center Loss function at the end of the structure. The new SoftMax-Center Loss function maps the model's output value to the [0, 1] interval during training. But the overall output sum is still 1. It is the probability value of the classification result needed in this paper.  Table 2 shows the whole structure of NVM.

## 4. Improvement and Design Based on SE-ResNet Module

### 4.1. Shortcut Connects and Bottleneck in ResNet

The results and discussion may be presented separately, or in one combined section, and may optionally be divided into headed subsections.

**Figure 7:**
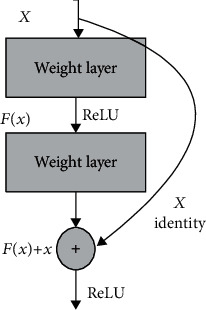
Structure diagram of shortcut connect.

It is not difficult to know from Figure 7 that shortcut connect transfers the top-level information in the CNN to the bottom layer of the network similar to a fast link, if we consider a 50-layer CNN as a process in which 50 people send notifications in sequence. In the transmission process of the warning to the 50 people, inevitably, the expression of the notice and the initial notice issued at the time of the 50th person are inconsistent due to some reasons. Shortcut connect is similar to a pager, and when it is passed from the first person to the second person, the third person is notified through the pager at the same time. This can effectively avoid the loss or miscommunication of information. The notification process from the first person to the third person is a complete residual connection module.

It is not difficult to understand why ResNet is superior to other CNNs in terms of convergence speed and classification accuracy. In each remaining module, shortcut connect makes convolutional layer learning difficult. Secondly, it guarantees efficient transfer of gradients.

**Figure 8:**
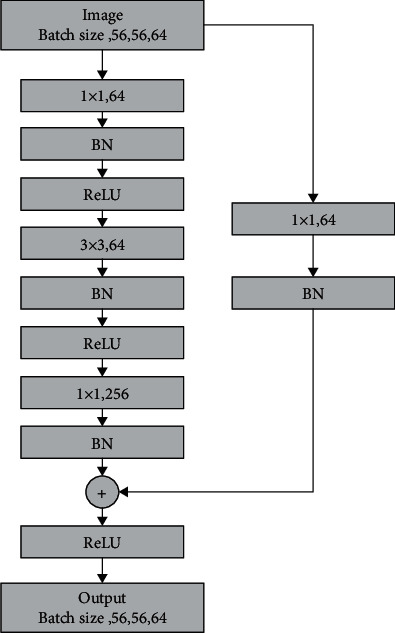
Schematic diagram of bottleneck structure.

Figure 8 is the bottleneck structure [15, 16]. As shown in this figure, we can see that the entire structure is firstly transformed from 256-dimensional input features to 64-dimensional by 1 × 1 convolution. Then, the feature extraction of a 3 × 3 convolution kernel is performed. Finally, the output dimension is restored to 256 dimensions through a 1 × 1 convolution kernel. The calculation of the entire operation parameter amount is analogous to the grouped convolution and depth separable convolution mentioned in the previous research, and the parameter amount can be reduced by 17 times.

### 4.2. Squeeze-and-Excitation Module

**Figure 9:**
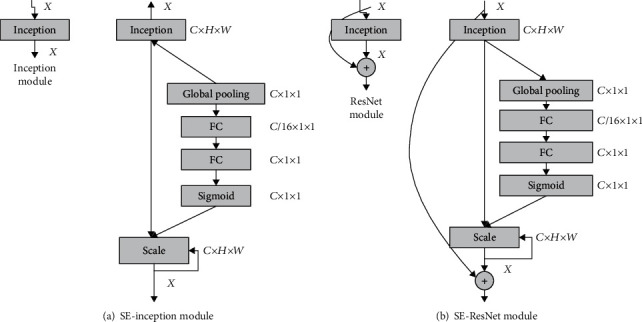
Schematic diagram of structure and composition of Squeeze-and-Exception module.

In the ILSVRC2017 computer vision competition, SeNet won the classification championship [17]. Its core module is Squeeze-and-Excitation module [18]. As shown in Figure 9, it first uses global average pooling as the channel for the Squeeze operation for features. Then, a Bottleneck structure composed of two fully connected layers is used to remove the correlation between channels. The feature dimension is first reduced, and the dimension is increased to achieve the purpose of reducing the parameter amount and the calculation amount.

### 4.3. Improvement of Squeeze-and-Excitation Module

The results and discussion may be presented separately, or in one combined section, and may optionally be divided into headed subsections.

According to the previous article, we improved the structure in the global pooling layer based on the Squeeze-and-Excitation module, grew the 1 × 1 convolution operation, and substituted the new loss function.

**Figure 10:**
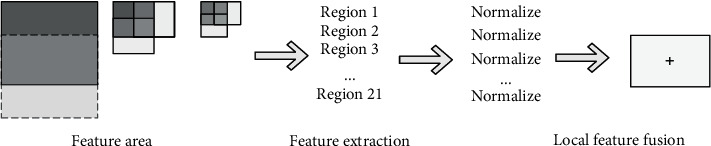
Schematic diagram of RMAC pooling process.

According to the introduction of the CNN pooling layer, the sliding window size of the general pooling layer is fixed. Based on this, RMAC pooling introduces variable sliding windows to pool features. As shown in Figure 10, we use three sliding window sizes. Each sliding window is Max-pooled for the feature map, and 20 local features can be obtained. Max pooling the entire feature map will also get a local feature. In total, we received 21 local features. Then, normalize and add the 21 local features to capture our final features. The advantage of this is that it can better extract the information between each channel and extract more features.

In each dataset, the system is easy to recognize some categories, but difficult for others. Besides, the number of these samples is different, so we choose the CNN classifier suitable for the moment. The number of samples in each category is prone to uneven proportions.

When a class has a large number of classifiers, the classifier can generally distinguish the class well. Conversely, when the number of classes is small, the performance of the classifier is not so good. In the conventional cross-entropy loss function, there is no distinction between the categories with a larger proportion of classifiers and the fewer categories. This will cause a waste of network resources. Because the system repeatedly learns those samples that have a good discrimination effect without focusing on training those samples with poor discrimination effects.

The focus loss function expression is shown in

(4) $$\begin{array}{c} \text{FL}\left(p_{t}\right)=-a_{t}{\left(1-p_{t}\right)}^{\gamma }\log\left(p_{t}\right). \end{array}$$

In Equation (4), (1 − *p*_*t*_) is the modulation index. It is used for the contribution rate of different sample categories to the loss function. It is not difficult to see that when *γ* = 0, the focus loss function becomes a conventional cross-entropy loss function [14]. In Equation (4),  *p*_*t*_ is the probability that the model predicts the sample category at the time of output. It divides the work according to the probability that our CNN predicts the sample category as the weight. Therefore, we use the focus loss function as a loss function to improve the network structure to reduce the training time of the improved model.

In the optimization of convolution, we use the idea of grouping convolution to replace all 1 × 1 convolution operations in the structure with the grouping convolution. However, we have not added channel shuffle in this network structure module. This is because the number of channels in the first three convolutions of the SE module itself is different. In the case of an inconsistent number of channels, packet convolution comes with the function of channel shuffle. And blindly adding channel rearrangement itself will increase the memory space occupied by the network structure and significantly reduce the network's applicability.

**Figure 11:**
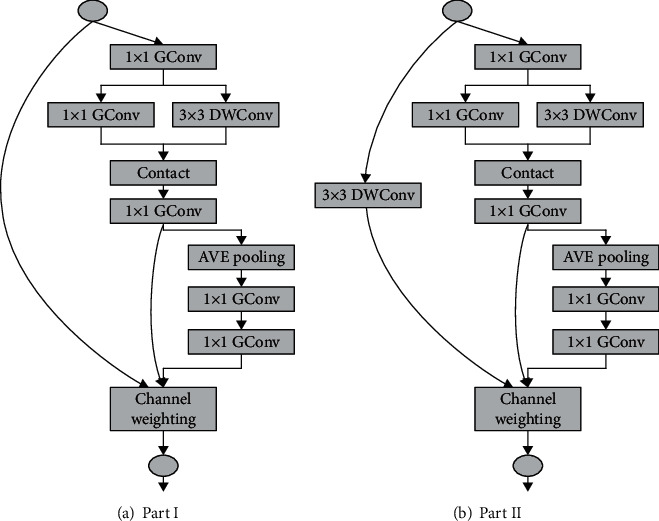
Schematic diagram of the improved SE module.

The improved Se module is shown in Figure 11. It can be seen that when the input feature map of the previous layer is passed to the module, it is divided into two branches in the module. One branch borrowed the idea of shortcut to cascade the input feature map and the output part. The other branch borrows the idea of a bottleneck to compress and change the feature channel.

When the input of the upper layer enters the RSE module, it will be divided into two parts. One part uses the branch of shortcut connects, which takes the input of the previous layer directly as the output. Another branch of the bottleneck part is used to reduce the dimensionality of the previous layer input and then input convolution kernels of different sizes for feature extraction. The RSE module uses a 1 × 1 convolution kernel and a 3 × 3 convolution kernel. This can ensure the diversity of receptive fields between different channels. The features through different convolution kernels are linked. The feature fusion of different receptive field feature channels is performed through a 1 × 1 convolution kernel. Based on the RSE module, the modules are cascaded. The entire network model is shown in Table 3.

## 5. Results and Analysis

**Figure 12:**
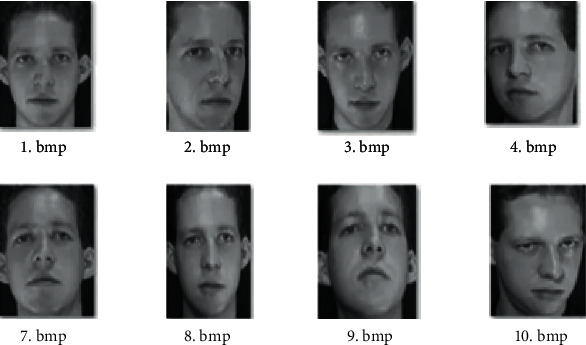
ORL dataset.

The ORL face dataset [19] was created in 1994 by the Olivetti Lab of the University of Cambridge, UK. There are 40 directories in the dataset. There are 10 facial expressions with different expressions stored in each directory. As shown in Figure 12, the pictures are saved in PGM format. Each image was acquired under different light and shadow, time, and facial features (open eyes, closed eyes, smile, and not smile). This experiment uses 300 of these pictures as the training set and 100 as the test set. The final output category is 40 (training on an Intel Xeon processor, Radeon Pro 580x graphics card, 32G memory MAC based on TensorFlow deep learning framework).

### 5.1. Effect of Packet Convolution on Experimental Accuracy and Network Quality

On the SqueezeNet, the SoftMax classifier at the end was also changed to a combination of SoftMax-Center Loss for monitoring. In the ORL dataset, this paper uses 4 groups on the number of NVM grouping convolutions.

As shown in Table 4, it can be seen that in the face recognition effect using the same classifier, the effect of grouping convolution on the classification accuracy has decreased, but it does not obviously mean that channel shuffle has completed the flow of information between channels. At the same time, after the introduction of grouping convolution, the parameter mount was effectively reduced by 33%. Reducing the model quality under the condition that the recognition effect has a limited impact indicates that the introduction of packet convolution in SqueezeNet is an effective model lightweighting strategy. However, because of the small number of training samples, the experimental accuracy is not very satisfactory.

### 5.2. Influence of Different Grouping Numbers on Structural Quality and Accuracy

Also on the ORL face dataset, the exponent of 2 can completely divide the dimension of the image input, so 2, 4, 8, and 16 grouping convolutions are used for training and testing, respectively. It can be seen from Table 5 that in the process of increasing the number of groups, the recognition accuracy is reduced by 1.2%, and the amount of parameters is correspondingly reduced by 13%. Under the comprehensive comparison, the effect is best when the number of current training sample groups is 4.

### 5.3. Realization and Effect Comparison of Lightweight Model

**Figure 13:**
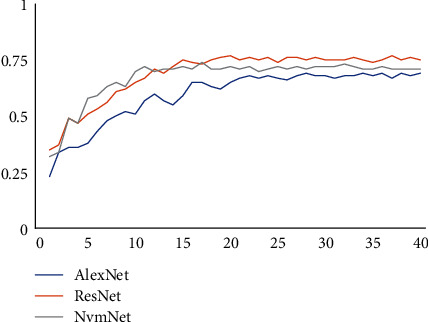
The training process of three different network structures on the ORL dataset.

This experiment trained 40 different epochs on three different network structures on the ORL dataset. As shown in Figure 13, the ResNet convergence speed of NVMNet and residual networks using packet convolution and batch normalization is faster than that of AlexNet using conventional convolution. Convergence can be done in about 15 epochs. The best classification accuracy on the ORL dataset is ResNet, NVMNet, and AlexNet. At the same time, it can be seen in the comparison of the parameter amount and calculation amount in Table 6, because SqueezeNet refers to the deep compression technology on the basis of the structure, so that the parameter is reduced to 1.24 trillion, NVMNet continues to reduce the number of parameters based on its original 4.6 trillion. In the case of using the same classifier at the same time, the accuracy does not decrease. It shows that the channel shuffle after grouping convolution is more effective.

As shown in Table 6, through comparison with several popular network structures, it can be seen that the AlexNet and VGG models of the conventional convolution mode have larger parameters than other lightweight models. This is related to the many parameters of their fully connected layer. The amount of ResNet parameters using the global average pooling layer is moderate, but because of its deep network structure, the amount of calculation is very large. The advantage of NVMNet in terms of parameter comparison is obvious. This greatly increases the application scenarios of the network model. Save application storage memory and computing costs. Model mobile portability is more excellent.

Compared with that of SqueezeNet, on the ORL dataset, the classification accuracy of NVMNet is decreased by 0.7%. However, the parameter amount was reduced by 33%. Therefore, NVMNet has a certain value in lightweight models.

### 5.4. Experimental Results and Data Analysis on the CIFAR-10 Dataset

**Figure 14:**
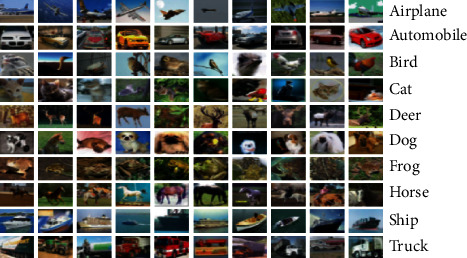
Overview of 10 categories and their respective pictures on the CIFAR-10 dataset.

The CIFAR-10 [20] dataset contains 60,000 color images with a resolution of 32 × 32. It contains a total of 10 categories: airplane, car, bird, cat, deer, dog, frog, horse, boat, and truck. There are 6000 pictures in each category. There are 10,000 test set pictures and 50,000 training set pictures. As shown in Figure 14.

We use the CIFAR-10 classic dataset for efficiency comparison and parameter comparison of our CNN model (training on an Intel Xeon processor, Radeon Pro 580x graphics card, 32G memory MAC based on TensorFlow deep learning framework). According to our hardware conditions and the requirements of the network structure, the network parameter learning rate is 0.01, the optimization strategy of the CNN is stochastic gradient descent (SGD), the learning rate change rate is 0.1, and the maximum number of iterations is 400000.

As shown in Table 7, it is obvious that without any other model-specific compression method, the R-SeNet size is 9.6 MB. Compared with the ResNet model, the weight of the network model is nearly 90 MB smaller, but the classification accuracy is almost the same, only 1.8% lower. If the model is further pruned by parameters or channel depth, the size of the network model can be further reduced to about 3 MB while ensuring accuracy.

**Figure 15:**
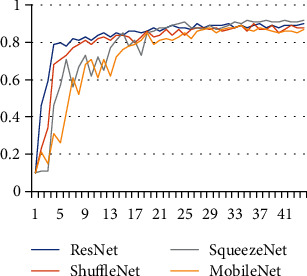
Comparison of training convergence of SqueezeNet, ResNet, ShuffleNet, and MobileNet.

We compare the convergence of R-SeNet with other popular lightweight CNN networks, such as MobileNet, ShuffleNet, and SqueezeNet for network convergence. The comparative convergence curve is shown in Figure 15. From the convergence curve, ResNet has faster convergence speed and less fluctuation than other CNNs in the initial training. The converged waveform is relatively stable. From the perspective of accuracy, the accuracy of the four network models is almost equal: SqueezeNet, ResNet, ShuffleNet, and MobileNet. Among them, ShuffleNet is still unstable after convergence, and the network fluctuates greatly, which may be related to the parameter optimization during CNN group convolution. Through verification, it can be seen that the improved ResNet has a certain improvement in convergence speed and model weight with a precision of only 1.3%.

Based on the comparison of different lightweight CNN structures, we compare the improved parts of ResNet separately to observe the impact of each method on the performance of the network structure. As shown in Table 8, it can be seen that after the RMAC pooling layer is improved, the network extracts more feature information, so the performance of the network structure is slightly improved by 0.8%. The change of the loss function has the greatest impact on structural performance, which has increased by 2.5% before and after the improvement. This may be due to the redundancy of the network during the calculation. The system repeatedly calculates many classifications that are better distinguished without targeted training. Therefore, with the introduction of the focus loss function, the entire CNN can dynamically allocate training resources along with the output probability value, allowing the system to learn more about those indistinguishable classes. After the introduction of packet convolution, network performance decreased slightly by 1.1%. It may be related to the information circulation between the packet convolution channels.

## 6. Conclusions

Berkeley and Stanford proposed SqueezeNet to reduce parameter dimensionality with the AlexNet and VGGNet models. The 1∗1 convolution kernel has been used to reduce the number of input channels of the network, thereby reducing the number of network parameters. It compresses the CNN, from hundreds of megabytes in original size, to 4.5 megabytes without affecting the accuracy of image classification. When deep compression network compression technology mentioned above is used, the amount of network parameters can be further reduced to about 0.5 trillion. The core of the model is a module called Fire Module. This paper introduces packet convolution to optimize the Fire Module. In order to solve the problem that the channel information does not circulate after the group convolution, a channel shuffle operation is added between the channels, and the classifier is optimized and improved according to the complexity of the face features.

The BCI system based on the convolutional neural network includes functional modules including visual triggering device, EEG acquisition device, EEG preprocessing module, classifier based on convolutional neural network, and classification result display. There are successive dependencies among various modules. The system will first receive the EEG signal data from the EEG collector and then filter and normalize it through EEG signal preprocessing and then use the data as the input of the EEG signal classifier. After these EEG data are recognized and classified by the classifier, the recognition results are displayed in the result output module of this system. Based on the predecessors, this paper makes lightweight improvements based on the Fire Module of the SqueezeNet convolutional network structure. This paper introduces batch normalization and the SoftMax-Center Loss classifier to improve the recognition accuracy and efficiency of the network structure under the face. In the case of refining the overall structure of the network, the classification effect on the ORL dataset has also improved. However, because the ORL dataset has relatively few training samples, data samples can be added for further verification in future experiments. The lightweight model structure has functional application scenarios. In the future, we plan to explore the feasibility of vision fields other than human faces, including applications in the BCI and BMI fields.

## Figures and Tables

**Table 1:** Comparison of parameters and calculations of traditional convolution and grouping convolution.

	Parameters	Calculations
Regular convolution	*C*_1_ × *C*_2_ × 9	2 × *C*_1_ × *h* × *w* × *H*_1_ × *W*_1_
Grouping convolution	(*C*_1_/*g*) × (*C*_2_/*g*) × 9 × *g*	2 × (*C*_1_/*g*) × (*C*_2_/*g*) × *h* × *w* × *H*_1_ × *W*_1_ × *g*
*R*	1 : (1/*g*)	1 : (1/*g*)

**Table 2:** NVMNet structure table.

Layer name	Output size (number of parameters)	*h*	*w*	*n*
Input	224 × 224 × 3			
Convolution layer	111 × 111 × 64			
Maximum pooling	55 × 55 × 64			
NVM2	55 × 55 × 64	16	16	64
NVM3	55 × 55 × 64	16	16	64
Maximum pooling 3	27 × 27 × 128			
NVM	27 × 27 × 256	32	32	128
NVM	27 × 27 × 256	32	32	128
Maximum pooling 5	13 × 13 × 256			
NVM6	13 × 13 × 384	48	48	192
NVM7	13 × 13 × 384	48	48	192
NVM8	13 × 13 × 512	64	64	256
Maximum pooling 9	6 × 6 × 512			
NVM11	6 × 6 × 512	64	64	256
NVM12	6 × 6 × 512	64	64	256
NVM13	6 × 6 × 512	64	64	256
Average pooling	1 × 1 × 512			
Full connection	Classification number			
SoftMax-Center Loss	Classification number			

**Table 3:** Parameters of each level of the R-SeNet network structure.

Layer	Input size (number of parameters)	Kernel size (number of parameters)	Stride
Input size	224 × 224	N/A	N/A
Convolution layer 1	112 × 112	7 × 7	2
Pooling layer 1	57 × 57	3 × 3	2
RSE.1	57 × 57	3 × 3	1
RSE.2	57 × 57	3 × 3	1
RSE.3	29 × 29	3 × 3	2
RSE.4	29 × 29	3 × 3	1
RSE.5	15 × 15	3 × 3	2
RSE.6	15 × 15	3 × 3	1
RSE.7	8 × 8	3 × 3	2
RSE.8	8 × 8	3 × 3	1
Pooling layer	1 × 1	N/A	Global

**Table 4:** Improved network quality nuclear accuracy comparison.

Structure	Parameter quantity (M)	Accuracy	
		Batch normalization	No-batch normalization
SqueezeNet	4.81	N/A	0.7125 ± 0.0004
NVM	3.27	0.7082 ± 0.0002	0.7016 ± 0.0013

**Table 5:** Influence of packet convolution of different groups on quality kernel accuracy.

Number of packets	Accuracy	Parameter quantity (M)
2	0.7142 ± 0.0021	3.31
4	0.7091 ± 0.0006	3.12
8	0.7069 ± 0.0009	3.01
6	0.7064 ± 0.0014	2.88

**Table 6:** Comparison of different network structures.

Structure	Parameter quantity (M)	Amount of computation
SqueezeNet	1.24	0.70
AlexNet	61.18	0.73
ResNet	11.69	3.49
ShuffleNet	1.32	0.32
Vgg-16	138	72
NVMNet	3.21	0.3

**Table 7:** Weight and resolution of CNN model on CIFAR-10 classic data.

CNN model	Network model weight (MB)	Network model compression ratio (%)	Network model accuracy (%)	Network model error distribution (%)
ResNet-50	98.1	N/A	95.1	N/A
ResNet-50@2.5	16.5	16.1%	93.1	-2.0
ResNet-50@.5	7.2	7.2%	93.1	-2.0
R-SeNet	9.6	10.1%	93.3	-1.8

**Table 8:** The influence of each compression thought segmentation on the accuracy of CNN.

	R-SeNet			
Grouped convolution		√	√	√
RMAC pooling			√	√
Focus loss function				√
CNN model accuracy (%)	88.1 ± 0.3	87.0 ± 0.5	87.8 ± 0.1	90.3 ± 0.1

## Data Availability

If necessary, you can contact the author of this article for relevant experimental data.

## References

[1] Phadikar S., Sil J., Das A. K. (2013). Rice diseases classification using feature selection and rule generation techniques. *Computers and Electronics in Agriculture*.

[2] Marty M. T. (2019). Eliminating artifacts in electrospray deconvolution with a SoftMax function. *Journal of the American Society for Mass Spectrometry*.

[3] Khishe M., Safari A. (2019). Classification of sonar targets using an MLP neural network trained by dragonfly algorithm. *Wireless Personal Communications*.

[4] Zheng D., Xiangqun L., Xu X. (2019). Vehicle and pedestrian detection network basedon lightweight SSD. *Journal of Nanjing Normal University (Natural Science Edition)*.

[5] Wu J., Yang Q., Zhongliang L. (2019). Face recognition based on improved squeezenet. *Science Technology and Engineering*.

[6] Guo Q., Quanli L., Wei W. (2019). Fast SqueezeNet algorithm with application in metro crowd density estimation. *Control Theory & Applications*.

[7] Pengchen B., Luo J., Weiwei C. (2019). Research on lightweight convolutional neural network technology. *Computer Engineering and Appications*.

[8] Yinhui Y., Zhengjin Z., Tao X. (2018). Improved SSD model based on SqueezeNet and its application. *China Traffic Informationization*.

[9] Dong Y., Yu J. (2018). Light-weight convolutional neural network SlimNet based on SqueezeNet. *Computer Applications and Software*.

[10] Pengcheng B., Luo J., Weiwei C. (2019). Lightweight convolutional neural network structure for mobile. *Information Technology and Network Security*.

[11] Camargo A., Smith J. S. (2009). Image pattern classification for the identification of disease causing agents in plants. *Computers and Electronics in Agriculture*.

[12] Qin L., Gong Y., Tang T., Wang Y., Jin J. (2019). Training deep nets with progressive batch normalization on multi-GPUs. *International Journal of Parallel Programming*.

[13] Kurita T., Otsu N., Sato T. A face recognition method using higher order local autocorrelation and multivariate analysis.

[14] Hong R., Tang J., Tan H.-K., Ngo C.-W., Yan S., Chua T.-S. (2011). Beyond search. *ACM transactions on multimedia computing communications and applications*.

[15] Hong R., Wang M., Gao Y. (2014). Image annotation by multiple-instance learning with discriminative feature mapping and selection. *IEEE Transactions on Cybernetics*.

[16] Liu C. L., Nakashima K., Sako H., Fujisawa H. (2003). Hand-written digit recognition: benchmarking of state-of-the-art techniques. *Pattern Recognition: The Journal of the Pattern Recognition Society*.

[17] Yu C., Tong T., Digen X. (2018). Joint supervision of center loss and softmax loss for face recognition. *Journal of Chongqing University*.

[18] He K. (2015). Zhang, Xiangyu, Ren, Shaoqing, et al. Spatial pyramid pooling in deep convolutional networks for visual recognition. *IEEE Transactions on Pattern Analysis and Machine Intelligence*.

